# American Dental Association

**Published:** 1874-10

**Authors:** 


### Proceedings of Societies.

### AMERICAN DENTAL ASSOCIATION.

CONTINUED FROM PAGE 398.

### 3d DAY, MORNING SESSION.

The Association met at half-past nine o'clock. The subject of Etiology, was passed.

### TREASURER'S REPORT.

The Treasurer, Dr. Goddard of Louisville, presented the Treasurer's report, from which it appears that there is a balance on hand of $213.23.

The salaries of the Secretary and Treasurer were ordered paid.

Dr. Cushing read an interesting paper prepared by Dr. I. Douglass, of Romeo, relating a case in which a patient under his care had been resuscitated from apparent death from anæsthetics by means of a galvanic battery.

### A RESOLUTION OF THANKS.

Dr. Shepard, of Boston, offered the following resolution, which was unanimously adopted:

*Resolved*, That the American Dental Association fully appreciates the promptness with which Dr. S. S. White responded to the requests of the dental profession to take the management of the suit for the ascertainment of the rights of the public in regard to the use of Vulcanite for dental purposes, and would respectfully request him to continue his valuable aid in carrying up the case for final adjudication by the Supreme Court of the United States, pledging him our moral and material support in defraying the expenses which may be incurred in the future.

Dr. Knapp, of New Orleans, read a report on the subject of Dental Education, prepared by Dr. A. S. McLean, of New Orleans. It treated of the value of dental colleges and of the advance made in dental education during the past score of years. He, however, criticised the management of some of the colleges. They do not require a sufficient amount of study, their course being about four months per year, and graduating a student in two years, after only eight months of study. This is not enough of study or practice, and the result is that the profession is suffering from dentists who have been graduated thus prematurely from these colleges. He advocated a longer term of study, a greater curriculum, the extension of the time for graduation to three years, of at least six or eight months study for each year, and that the graduate receive his degree only after passing a thorough critical examination on the principles of the science and the practice of dentistry. Some such course as this he claimed was demanded for the interests of the profession.

Dr. Keely read a paper on the same subject, taking the ground that a broader scope of culture should be required from the students in this profession. He urged that each student should be placed under the instruction of competent preceptors, who should keep before them the demands for high qualifications for their profession. Instead of establishing various dental colleges at many places it would be better to concentrate all efforts at three or four different points. Four dental colleges will be sufficient for the country for years to come, one in the East, one in the West, one in the South, and in due course of time one on the Pacific coast. He thought that means should be provided for giving larger salaries to the professors of dental colleges and this could be done, and the best of instructors could be secured by this policy of concentrating the colleges and securing for them competent endowments.

Dr. John Allen said that in addition to the education that is imparted to students at the dental college, they have a second education which they give to themselves, and which has been called a self-education. dental colleges have not always done their duty with their graduates, and many have graduated who are unfit to enter the profession. After graduating the student should still pursue a system of self-education, not only acquiring knowledge of his profession from books, but learning from all sources that he can whatever may be of advantage to him in the practice of his profession.

Dr. Walker, of New Orleans, hoped that the dental colleges would go further than they have yet done. When we see dentists breaking down every year over the breath of their patients, it is very evident that something more should be learned by the dentists for their own good.

Dr. Taft, of Cincinnati, said great care should be exercised in the training of students, and also in reference to their preliminary education, their desire for, and devotion to study, and their aptness to learn. The student should acquire a knowledge of the fundamental principles of medicine, before taking up the study of dentistry, and also should have a good literary education as a ground work. He should be made to understand that he must have a thorough training in his profession before entering upon it, and that it will require from four to seven years to acquire it. The experience of colleges to-day is that students importune to be graduated in a single year or a year and a half, and too frequently these importunities are complied with. Therefore, some of the colleges are to blame for the short-comings of their students. In the Ohio college, he said, the students from Michigan come better prepared than from any other State. The value of a preliminary education could not be over estimated. Local and State dental societies are taking up the matter of dental education, and this association ought to make a pronouncement on this subject. This body should take a high stand, and all the local societies should be brought up to it. If a clear announcement is made on this subject in this body no college in the country could afford to disregard it.

Dr. G. B. Thomas, of Detroit, said that no dentist should advise a student that he can become fully prepared to practice dentistry in less than seven years study. He hoped to see the day when a graduate of a dental college will be as capable of practicing medicine as he is dentistry, for dentistry is but a specialty of medicine. He advocated the highest preliminary education and qualification for the student entering upon the practice of dentistry.

Dr. Stockton, of New Jersey, said the great difficulty is that men are in too much haste to learn the art, and called to mind the case of a man who, after studying for nine months, set himself up for a grandfather in the profession. He advocated a system of examination before a board of censors of all men before they are admitted to practice, and laws should be made in every State to that end.

Dr. Bogue also urged that the association take a decided stand upon an advanced course of study, for then the colleges would be obliged to advance their standard.

Dr. Butler, of Cleveland, thought that diplomas from dental colleges are too cheap, students often enter colleges after a few months in some dentist's office, and are graduated as dentists in the course of a year or two. The colleges should be reduced to two or three, with the best men as professors, and with a high standard of qualification necessary for admission. Dr. Butler offered a resolution in this connection, that it is the opinion of the American Dental Association that the time has fully come when degrees should not be conferred by dental colleges upon any student who has attended but one course of lectures.

Dr. Shepard thought no college should be allowed to graduate in one term. The time will soon come when two full courses of eight months each must be adopted by every college of good standing in the country

Dr. Morgan said the medical college connected with the Virginia University was endowed with a sufficient amount to keep the best of professors, and that diplomas are granted only upon real merit. He hoped to see dental colleges so endowed, and students who graduate should do so only after passing a thorough and qualified examination. The Maryland

Dental Society, he said, was the first society to require that students should have at least two years course in a college.

Dr. Wetherbee, of Boston, said that by far the largest portion of the community who required dental services were persons of small means, and they were accustomed to call upon dentists who would perform the work required for the least sum. There is a large number of dentists that have not had the required amount of training and study in their profession. They have gone out as grandfathers of the profession after three months of practice. There is a class of dentists who take students into their offices and who have given them little or no instruction in the mechanical part of dentistry, as to the method of filling teeth. Others require young men to pay a certain sum for three years and even then never show them how to fill a tooth. He held that a dentist should take a student for three years and after that time require him to take a course of lectures for three years. He urged as a means of elevating the profession, the amending of the constitution so that no dentist shall become a member of the association unless he is a graduate of a college.

Piof. McQuillen, of Philadelphia, queried whether all these criticisms of dental colleges were just, and suggested that many who criticise these institutions do absolutely nothing toward endowing dental colleges. He believed that the grade of dentists is being elevated instead of being lowered, and said that many graduates of dental colleges have gained a wide reputation at home and abroad for their work and for essays on various scientific subjects. He believed that the number of colleges ought not to be diminished, for young men will go to colleges where they are taught the most for their time and money. No college eould take more than one hundred pupils and give to all the students the privileges required by them. He could see no advantage in connecting a dental with a medical college.

Dr. Crouse, of Chicago, thought that there had been too much said of the superiority of graduates of dental colleges. His experience, which he said was large, was that some of the worst practitioners and greatest mounte-banks in the profession were those who had graduated from dental colleges. He thought there were altogether too many dental colleges in the country, and that they were too poor. If he should take a student into his office to learn dentistry, after giving him all the instruction he could, he would not send him to any dental college in the country, but would send him to some good medical college. There is no need of so many dental colleges all over the country. Two or three are enongh, and until he sees students come out better dentists from dental colleges, he will not advise any student to attend one, but would prefer that they should graduate from a good medical college and then pursue their studies in a dentist's office.

Dr. Atkinson, of New York, said there is too much involved in dental education to speak of it in the ten minutes allowed him. He was sorry to see a distinction made between dental and medical education. A medical is the basis of a dental education. If we could but recognize the difficulties that arise in the matter of dental education we might do something about it, but we can not. If there must be diplomas from colleges, by all means let them say what they mean. There are dentists in this association who have received diplomas and deserved to receive them, but they state a falsehood upon them. They are men who sought the truth in dental science, have worked to obtain it, and they deserve their diplomas. He said that a dentist's mistakes, are more easily observed than a physician's. A dentist who breaks a man's jaw or injures him in any way, because of his lack of skill, will be held up to public scorn and contempt, as not knowing how to practice his profession, but a physician's blunders are usually buried with his patient in the grave. He held the profession of dentistry above that of medicine. He did not believe in the education of the masses together. Only those who are all eager and hungry to learn can be taught properly together. Personal, individual contact of the teacher and student is the proper method of teaching.

### APPLIANCES.

Dr. George L. Field, from the Committee on Appliances, reported the name of a number of instruments, machines, etc., that had been examined by the committee, and the value of each to the profession.

The association then adjourned until half-past two o'clock.

### 3d DAY, AFTERNOON SESSION,

The Association met according to adjournment. The discussion on "Operarive Dentistry" was resumed by Dr. Palmer, of Syracuse, who spoke of the use of galvanism in opperative dentistry, Dr Knapp,of New Orleans, also spoke on hæmorrhage, after a tooth is extracted, and its remedy.

Dr. Atkinson, of New York, thought that in the discussion to much had been said about the mere mechanism of the work of filling the teeth, and not enough of the principles of the science. The best of results, he thought, could be secured from the use of the rubber dam. The kind of gold used, he did not consider of any special importance, so long as the work was done well, and the best thing a dentist can do is to do the best kind of work.

The general subject was also further discussed by Drs. Taft, Walker, Webb, Forbes, Thomas, Rehwinkle, Allport, and Hunter, principally upon the treatment of diseased pulp, after which the subject was passed.

### DENTAL APPLIANCES.

Dr. George L. Field, chairman of the Committee on Dental Appliances, read a report upon the various new appliances for the use and benefit of the dental profession in the filling and extracting of teeth, the committee making favorable report on such as, in their opinion, deserved it. The subject was passed.

### ETIOLOGY.

The subject of etiology was again resumed, and Dr. J. H. McQuillen, made a report illustrating the subject with diagrams on the black board, and showing the presence of parasites in the teeth, and their action in producing decay. He was followed by Dr. Noel, of Nashville, Tenn., after which the meeting adjourned until half past seven o'clock.

3d DAY, EVENING SESSION.

On motoin of Dr. G. Crouse the association proceeded to select a place for the next annual meeting of the association on the first Tuesday of August, 1875, and chose Niagara Falls.

The association then proceeded to election of officers for the ensuing year, with the following result:

President, M. S. Dean, Chicago.

Vice Presidents, George W. Keely, Oxford, O., and James S. Knapp, of New Orleans.

Corresponding Secretary, Dr. George L. Field, of Detroit.

Recording Secretary, Dr. C. S. Smith, of Springfield, Ill.

Treasurer, Dr. W. H. Goddard, of Louisville Ky.

Executive Committee, for one year, C. H. Cushing, of Chicago; L. D. Shepard, Boston: H. A. Smith, Cincinnati; George L. Field, George R. Thomas, Detroit.

For two years, Dr. A. L. Brockway, of Brooklyn; Dr. G. C. Dayboll, Buffalo; S. B. Palmer, Syracuse N, Y.

The association then adjourned.

4th DAY, MORNING SESSION.

The Association met according to adjournment. Dr. Stockton, of New Jersey, gave notice that he would bring in an amendment to the constitution providing for a change in the manner of electing officers of the Association to be acted on next year.

Dr. Shepard also offered a resolution of thanks to the reporters of the morning papers, for correct reports of the proceedings of the Association; to the Detroit dentists for courtesies extended; to the city government for use of council chamber, and to the hotel propriecors for efforts made to render the sojourn of members here pleasant. Adopted.

Dr. Goddard, of Louisville, called attention to the fact that the expenses of the association exceeded the reciepts and that a large amount of these came from printing 500 copies of the proceedings. Less than 100 of these are needed for the members, some fifty more are sent to different societies, and the remainder are in the hands of the Secretary. A motion to send these extra copies of the proceedings to various local societies and dentists who are willing to pay the postage on them, was adopted as was also another motion to have only 250 copies printed the coming year.

### STANDING COMMITTEES.

The Executive Committee announced the following standing Committees:

On Physiology, Drs. J. H. McQuillen, E. S. Gaylord, J. B. Walker.

On Pathology, Drs. H. Judd, L. D. Shepard, J. S. Knapp.

On Histology and Microscopy, Drs. J. Taft, E. D. Swayne, M. H. Jackson.

On Chemistry, Drs. H. A. Smith, S. B. Palmer, J. S. Cassiday.

On Therapeutics, Drs E. A. Bogue, W. O. Kulp, C. C. Carroll.

On Operative Dentistry, Drs. G. H. Cushing, C. S. Stockton, S. H. McCall.

On Mechanical Dentistry, Drs. F. H. Rehwinkle, J. F. Canine, J. Johnston.

On Dental Education, Drs. G. W. Keely, A. H. Brockway, S. Welchens.

On Dental Literature, Drs. J. S. Knapp, L. G. Noel, C. D. Cook.

On Etiology, Drs. H. S. Chase, D. C. Hawxhurst, C. S. Smith.

On Prize Essays, Drs Forbes, G. L. Field, R. B. Donaldson.

### DENTAL EDUCATION.

The subject of dental education was then resumed and was further discussed by Drs. Rehwinkle and Horton, of Ohio, both of whom spoke in advocacy of the system of dental colleges, and defended the Ohio Dental College against an attack that had been made upon it.

Dr. Homer Judd, of St Louis, said the dentists are a specialty of the medical profession, but they have no right to complain of the treatment they have received from the medical profession. He believed that the great body of educated physicians acknowledge and extend the right hand of fellowship to the dentists, the same as they do to the oculists and specialties in the profession, who are properly educated to the professsion. If dentists wish to obtain a knowledge of the medical profession, they must get it from the best sources, and these are the medical colleges. He believed that the dental colleges of the land had done a great amount of good. That all had done their duty he would not say. It would be strange if they had. Certain schools are kept up for the benefit of those who are connected with them as professors.

Dr. Bogue offered the following resolutions:

*Resolved*, That it is the sense of the Association that no dental student should be graduated by any dental college without at least three years instruction, including private pupilage and college instruction. The latter should in no case embrace less than two regular full courses.

*Resolved*, That the Association suggest to the different colleges of this country to appoint a common examining committee, consisting of five members, three to be a quorum, none of whom shall be in connection with any dental college, whose duty it shall be to examine all applicants for graduation and decide upon the same.

The resolutions, together with that previously offered by Dr. Bogue, were laid on the table until next year.

Dr. Judd offered the following:

*Resolved*, That this Association recommend to all local societies the adoption of rules prohibiting their members from taking students for a less period than three years or for such time as will complete a three years' pupilage.

This resolution was also tabled for a year.

Dr. Allport gave notice that he would, at the next session of the Association, bring in an amendment to the constitution requiring that one of the requisites for membership in the Association in any dentist who shall begin practice from and and after this time shall be, that he be a graduate either of a medical or dental college.

Dr. Knapp offered a resolution of thanks to the members of the Michigan Dental Association for their efforts in contesting the validity of the Cummins' Vulcanite Patent.

The resolution was discussed by Drs. Spelman and Horton setting forth a statement of the case, with assurance from distinguished attorneys that the dentists will gain their case.

The resolution was adopted.

Dr. S. B. Palmer, from a committee appointed to prepare a circular containing such information as the public need respecting the care of teeth, asked for further time in which to perfect their report. Granted.

Dr. Keely, from the Committee on the Barnum Memorial Fund, asked further time to report. Granted.

### MECHANICAL DENTISTRY.

Dr. M. S. Dean, of Chicago, read the report of the Committee on Mechanical Dentistry. The report embraced a a criticism of various appliances which have been introduced into the mechanical department of dentistry in the past year, and various modes of treatment. The report was ordered placed on file.

### MISCELLANEOUS.

The subjects of histology and microscopy, of therapeutics, and of local societies were passed, no reports being ready from any of the committees.

The Association then adjourned *sine die.*

